# Loss of *CDKN2A* at chromosome 9 has a poor clinical prognosis and promotes lung cancer progression

**DOI:** 10.1002/mgg3.1521

**Published:** 2020-11-06

**Authors:** Wei Liu, Congwen Zhuang, Tengfei Huang, Shengsheng Yang, Meiqing Zhang, Baoquan Lin, Yi Jiang

**Affiliations:** ^1^ Department of Respiratory and Critical Care Medicine The 900th Hospital of Joint Logistic Support Force Fuzhou Fujian China; ^2^ Department of Thoracic Surgery The 900th Hospital of Joint Logistic Support Force Fuzhou Fujian China; ^3^ Department of Hepatobiliary Surgery The 900th Hospital of Joint Logistic Support Force Fuzhou Fujian China

**Keywords:** CDKN2A, copy number variation, lung cancer, MTAP

## Abstract

**Objective:**

This study aimed to identify critical genes involved in the tumor biology of lung cancer via datamining of The Cancer Genome Atlas (TCGA) with special focus on gene copy number variation.

**Methods:**

Genomic deletion and amplification were analyzed with cBioportal online tools. Relative expression of Cyclin Dependent Kinase Inhibitor 2A (*CDKN2A*) was analyzed by both real‐time polymerase chain reaction (PCR) and Western blot. The abundance of methylthioadenosine phosphorylase (*MTAP*) and epithelial‐mesenchymal transition markers were analyzed by real‐time PCR. Cell proliferation was determined by cell counting kit‐8 method and cell viability was measured with 3‐(4,5‐dimethylthiazol‐2‐yl)‐2,5‐diphenyltetrazolium bromide assay. The cell migration and invasion were measured with transwell chamber assay, and migrative capacity was further evaluated by wound healing assay.

**Results:**

We found the frequent loss of CDKN2A was associated with its downregulation in lung cancer, and siRNA‐mediated *CDNKN2A* knockdown significantly stimulated cell proliferation, invasion, and migration. Mechanistically, we unraveled that MTAP, which was positively correlated with CDKN2A, predominantly mediated the antitumoral function of CDKN2A in lung cancer.

**Conclusion:**

Our study consolidated the involvement of CDKN2A‐MTAP signaling in the context of lung cancer.

## Graphical abstract

The frequent loss of CDKN2A associated with its down‐regulation in lung cancer. The study highlighted the importance of CDKN2A‐MTAP signaling in lung cancer.

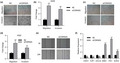

## 1 INTRODUCTION

Lung cancer is one of the most common human malignancies globally (Barnett, 2017). Based on Cancer Statistics 2012, there were 1.8 million new cases diagnosed and 1.6 million deaths claimed by this disease (Siegel et al., 2012), making lung cancer the most common cancer‐related death in men while the second in women only after breast cancer. Histologically, lung cancer is mainly categorized into two types: small cell lung carcinoma (SCLC) and non‐small cell lung cancer (NSCLC). Long‐term tobacco smoking contributes to the huge majority of morbidity of lung cancer (Lin et al., 2008), and other recognized risk factors include combination of genetic abnormalities (Dai et al., 2019), radon gas, asbestos, and other forms of air pollution. Therefore, avoidance of smoke and air pollutant constitutes the first line of prevention (Simon, (2016)). Clinical treatments for lung cancer are heavily dependent on molecular subtype, progression stage and the overall personal health status, and include surgery, chemotherapy, radiotherapy, targeted therapy, and emerging immunotherapy (Hirsch et al., 2017; Liu et al., 2020). Despite of the advances in therapeutic interventions, the overall survival of lung cancer is still relatively unsatisfactory with the 5‐year survival rate of around 20% in the United States.

Activation of oncogenes and/or inactivation of tumor suppressor genes have been long acknowledged as the initiating events in the tumorigenesis of lung cancer. The environmental carcinogens are frequently identified to be involved in the generation of mutations in these genes. K‐ras proto‐oncogene mutations are reported to account for 10%–30% cases of lung adenocarcinomas (Ferrer et al., 2018), and 40% of NSCLCs are characterized with echinoderm microtubule associated protein‐like 4/anaplastic lymphoma kinase tyrosine kinase fusion gene (Sasaki et al., 2010). The epidermal growth factor receptor (EGFR) which signals cell proliferation and angiogenesis is commonly and aberrantly over‐activated in NSCLC, which in general shows favorable response to EGFR‐inhibitor treatments (Tu et al., 2017). In addition, epigenetic mechanisms have also critically contributed to lung cancer via DNA methylation, histone modification, and microRNA network modulation. In this case, Cyclin Dependent Kinase Inhibitor 2A (*CDKN2A*, OMIM association number 600160), the essential cell cycle regulating factor, is increasingly recognized to be involved in the pathological process of lung cancer. The first two studies identified deletion of CDKN2A in multiple human cancers in 1994 (Kamb et al., 1994; Nobori et al., 1994), and subsequent investigation uncovered that CDKN2A loss was restricted to a subpopulation of lung cancers with intact retinoblastoma (RB) (Otterson et al., 1994), which was further disclosed as reciprocal *RB* inactivation and *CDKN2A* expression in primary lung cancers and cell lines (Shapiro et al., 1995). In the following study, Merlo et al. identified that 5′CpG island methylation was associated with suppressed transcription of *CDKN2A* in human cancers (Merlo et al., 1995), which was then found to be frequently associated with aberrant inactivation of *CDKN2A* (Herman et al., 1995). The study performed by Belinsky et al. suggested that aberrant methylation of *CDKN2A* was an early event in lung cancer patients and served as a potential biomarker for diagnostic purpose (Belinsky et al., 1998). Here, we retrieved the publicly available database and unraveled the causal relationship between genomic deletion and downregulation of *CDKN2A* in lung cancer patients, which clinically associated with unfavorable prognosis. The antitumoral properties of *CDKN2A* was uncovered in cell culture. Most importantly, here, we further identified a positive correlation between *CDKN2A* and Methylthioadenosine Phosphorylase (*MTAP*, OMIM association number, 156540) in lung cancer. The suppressed expression of MTAP predominantly contributed to the oncogenic signaling in CDKN2A‐deficient lung cancer.

## 2 MATERIALS AND METHODS

### 2.1 The Cancer Genome Atlas (TCGA) data analysis

Genomic deletion/amplification, copy number variation of *CDKN2A*, and survival curve in terms of CDKN2A deletion status in lung cancer patients were analyzed against TCGA datasets containing multiple subtypes of lung cancer, such as NSCLC, SCLC, and mesothelioma, using the cBioportal algorithm (http://www.cbioportal.org).

### 2.2 Cell culture

The human lung cancer cell lines A549 and H322 were purchased from the American Type Culture Collection (NY, USA) and maintained in RPMI‐1640 medium containing 10% of fetal bovine serum (FBS, Gibco, MA, USA) and 1% of penicillin/streptomycin (Hyclone, MA, USA). All cells were cultured in a humidified CO_2_ incubator (5%). Cell lines were verified using the short tandem repeat analysis. Mycoplasma contamination was regularly monitored by PCR method.

### 2.3 Real‐time polymerase chain reaction (PCR)

TRIzol (Invitrogen, MA, USA) was used to extract total RNA from both A549 and H322 cells (1 ml/well of 6‐well plate). RNA quality was analyzed with BioAnalyzer 2100 (Agilent, CA, USA) and quantified with NanoDrop 1000 (Thermo Fisher, MA, USA). The QuantiTect Reverse Transcription Kit from Qiagen (Hilden, Germany) was employed for cDNA preparation. Quantitative PCR was performed on 7500 Fast Dx Real‐Time PCR Instrument (Applied BioSystems, CA, USA) with QuantiTect SYBR Green PCR Kit (Qiagen). Relative expression of genes was calculated using the 2^−∆∆Ct^ method. The primers used were listed as below:

*CDKN2A* F: 5′‐ATGGAGCCTTCGGCTGACT‐3′

*CDKN2A* R: 5′‐GTAACTATTCGGTGCGTTGGG‐3′

*MTAP* F: 5′‐ACCACCGCCGTGAAGATTG‐3′

*MTAP* R: 5′‐GCATCAGATGGCTTGCCAA‐3′

*CDH1* (OMIM association number, 192090) F: 5′‐CGAGAGCTACACGTTCACGG‐3′

*CDH1* R: 5′‐GGGTGTCGAGGGAAAAATAGG‐3′

*TJP1* (OMIM association number, 601009) F: 5′‐CAACATACAGTGACGCTTCACA‐3′

*TJP1* R: 5′‐CACTATTGACGTTTCCCCACTC‐3′

*OCLN* (OMIM association number, 602876) F: 5′‐ACAAGCGGTTTTATCCAGAGTC‐3′

*OCLN* R: 5′‐GTCATCCACAGGCGAAGTTAAT‐3′

*ZEB1* (OMIM association number, 189909) F: 5′‐GATGATGAATGCGAGTCAGATGC‐3′

*ZEB1* R: 5′‐ACAGCAGTGTCTTGTTGTTGT‐3′

*FN1* (OMIM association number, 135600) F: 5′‐CGGTGGCTGTCAGTCAAAG‐3′

*FN1* R: 5′‐AAACCTCGGCTTCCTCCATAA‐3′

*EZH2* (OMIM association number, 601573) F: 5′‐AATCAGAGTACATGCGACTGAGA‐3′

*EZH2* R: 5′‐GCTGTATCCTTCGCTGTTTCC‐3′

*GAPDH* (OMIM association number, 138400) F: 5′‐GGAGCGAGATCCCTCCAAAAT‐3′

*GAPDH* R: 5′‐GGCTGTTGTCATACTTCTCATGG‐3′

### 2.4 Western blot

Cells were lysed in ice‐cold radioimmunoprecipitation assay lysis buffer and cell debris was removed by centrifugation. Proteins were resolved with 10% of sodium dodecyl sulfate‐polyacrylamide gel electrophoresis and followed by transferring onto polyvinylidene difluoride membranes. Membranes were incubated with rabbit anti‐CDKN2A (1:1000, ab211542, Abcam, Cambridge, UK) and anti‐GAPDH antibodies (1:2000, ab9485, Abcam) overnight at 4℃, and then, hybridized with horseradish peroxidase‐conjugated goat anti‐rabbit secondary antibody (1:3000, ab6721, Abcam) at room temperature for 1 hour. Blots were visualized using enhanced chemiluminescence kit (ECL, Millipore, MO, USA) on ChemiDoc Imaging Systems (Bio‐Rad, CA, USA).

### 2.5 2,5‐diphenyl tetrazolium bromide (MTT) assay

MTT Assay Kit (ab211091, Abcam) was employed to determine the viability in exponentially growing cells. The indicated cells were plated in 6‐well plates and cultured overnight for attachment. The 1:1 mixture of serum‐free media and MTT reagent (100 μl) was then replaced and followed by 3 h of incubation at 37℃. 150 μl of MTT solvent solution was then replaced and followed by 15 min of incubation at room temperature on a shaker. Absorption at 590 nm was recorded on a microplate reader (Berthold Technologies, Bad Wildbad, Germany).

### 2.6 Cell counting assay

Cell counting kit‐8 (CCK‐8, Dojindo, Dalian, China) was used to measure cell proliferation following the manufacturer's instructions. Cells were first seeded in 96‐well plates (1500 cells/well) and cultured for 24 h. About 10 μl/well of reagent solution was added and followed by incubation for 2 h at 37℃. Absorption was recorded with a microplate reader (Berthold Technologies).

### 2.7 Cell invasion, migration, and wound healing assays

Cell invasion and migration were evaluated with Boyden chambers (BD Biosciences, NJ, USA) with or without Matrigel precoating, respectively. Wound scratch was created with sterile tips on 6‐well plates. Gap closure was continuously monitored for 24 h. All experiments were performed in triplicate and result was normalized to cell numbers.

### 2.8 Statistical analysis

Results are presented as means ±standard deviation (SD). All the experiments were repeated as biological replicates for at least three times. Differences between groups were estimated with Student's *t* test, one and two‐way ANOVA analysis with a post hoc test where appropriate. *p* values <0.05 were considered as statistically significant.

## 3 RESULTS

### 3.1 Loss of CDKN2A in lung cancer

**FIGURE 1:**
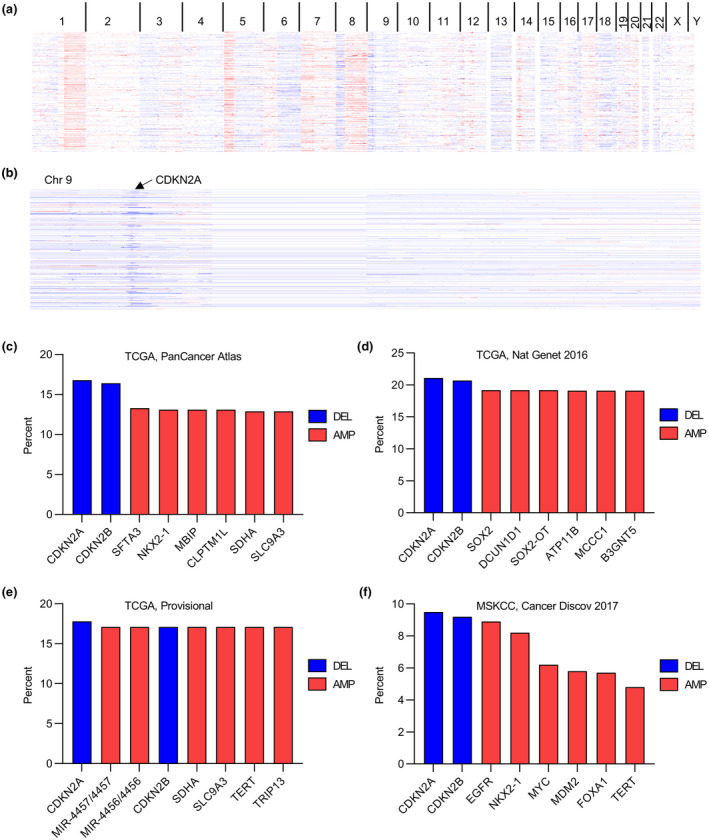
Loss of CDKN2A in lung cancer. (a) Heatmap represents the gene copy number alteration in the whole genomic of lung cancer patients in TCGA datasets. (b) Heatmap represents the gene copy number alteration in the chromosome 9 of lung cancer patients in TCGA datasets. (c–f) Genes with the highest frequency of copy number alteration in TCGA lung cancer datasets of PanCancer Atlas (c), Nat Genet 2016 (d), Provisional (e), and Cancer Discov 2017 (f). All the TCGA datasets were analyzed by cBioportal (http://www.cbioportal.org)

We first analyzed the gain and loss of genomic fragments in lung cancer at genome‐wide level in the TCGA database with the cBioportal algorithm (Figure 1a), and uncovered a characteristic depletion of genomic region in chromosome 9 across the *CDKN2A* coding sequence (Figure 1b). We further demonstrated the highest frequency of *CDKN2A* depletion among four datasets in lung cancer patients including PanCancer Atlas (Figure 1c), Nat Genet 2016 (Figure 1d), Provisional (Figure 1e), and Cancer Discov 2017 (Figure 1f), which indicated the universal genomic depletion of *CDKN2A* and its essential roles in the tumorigenesis of lung cancer. Therefore, our following investigation focused on the importance of CDKN21 in this disease via analyzing both clinical and in vitro data.

### 3.2 Loss of *CDKN2A* correlated with poor survival outcomes in lung cancer

**FIGURE 2:**
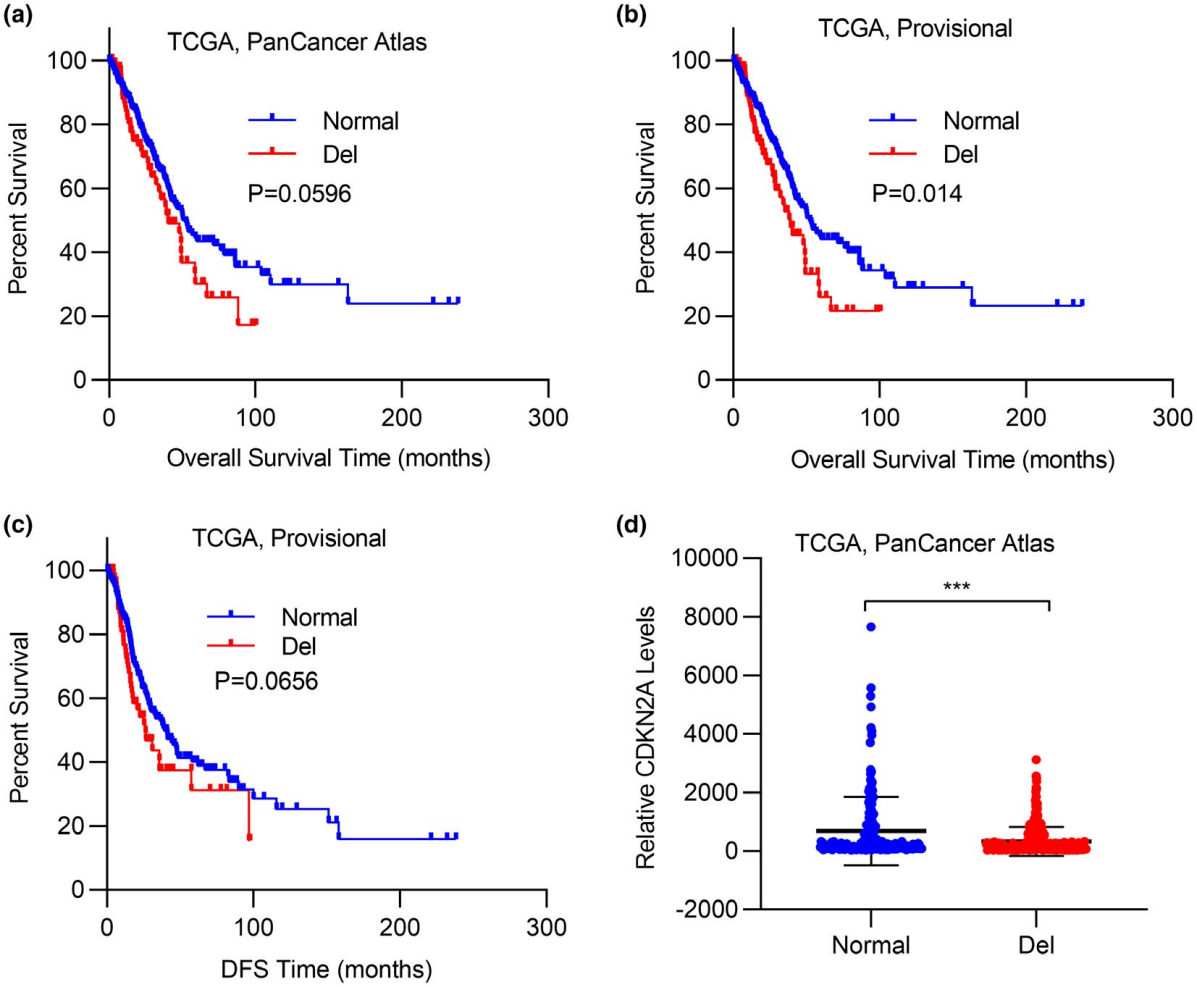
Loss of CDKN2A correlated with poor survival outcomes in lung cancer. (a) Kaplan–Meier plots of overall survival in lung cancer patients stratified according to their CDKN2A genomic status in TCGA PanCancer Atlas datasets. (b) Kaplan–Meier plots of overall survival in lung cancer patients stratified according to their CDKN2A genomic status in TCGA Provisional datasets. (c) Kaplan–Meier plots of disease‐free survival in lung cancer patients stratified according to their CDKN2A genomic status in TCGA Provisional datasets. (d) The CDKN2A expression levels in lung cancer patients with or without CDKN2A deletion in TCGA PanCancer Atlas datasets. Data are shown as mean ± SD. *p < 0.05; **p < 0.01; ***p < 0.001; ns, not significant

We next investigated the potential linkage between *CDKN2A* depletion and clinical outcomes in lung cancer patients via analyzing the survival curve. As shown in Figure 2a, depletion of *CDKN2A* significantly associated with poorer survival. And consistent observation was noticed in the “TCGA, Provisional” dataset as well (Figure 2b). Likewise, *CDKN2A* deficiency indicated a poorer disease‐free survival in the “TCGA, Provisional” dataset (Figure 2c). These results suggested the potent tumor suppressor role of *CDKN2A* in lung cancer, especially in tumorigenesis and tumor progression. Further analysis uncovered the relatively low expression of *CDKN2A* transcript in lung cancer patients with *CDKN2A* depletion (Figure 2d), which highlighted the important contribution of genomic loss to the downregulation of *CDKN2A*.

### 3.3 Loss of *CDKN2A* promoted lung cancer proliferation

**FIGURE 3:**
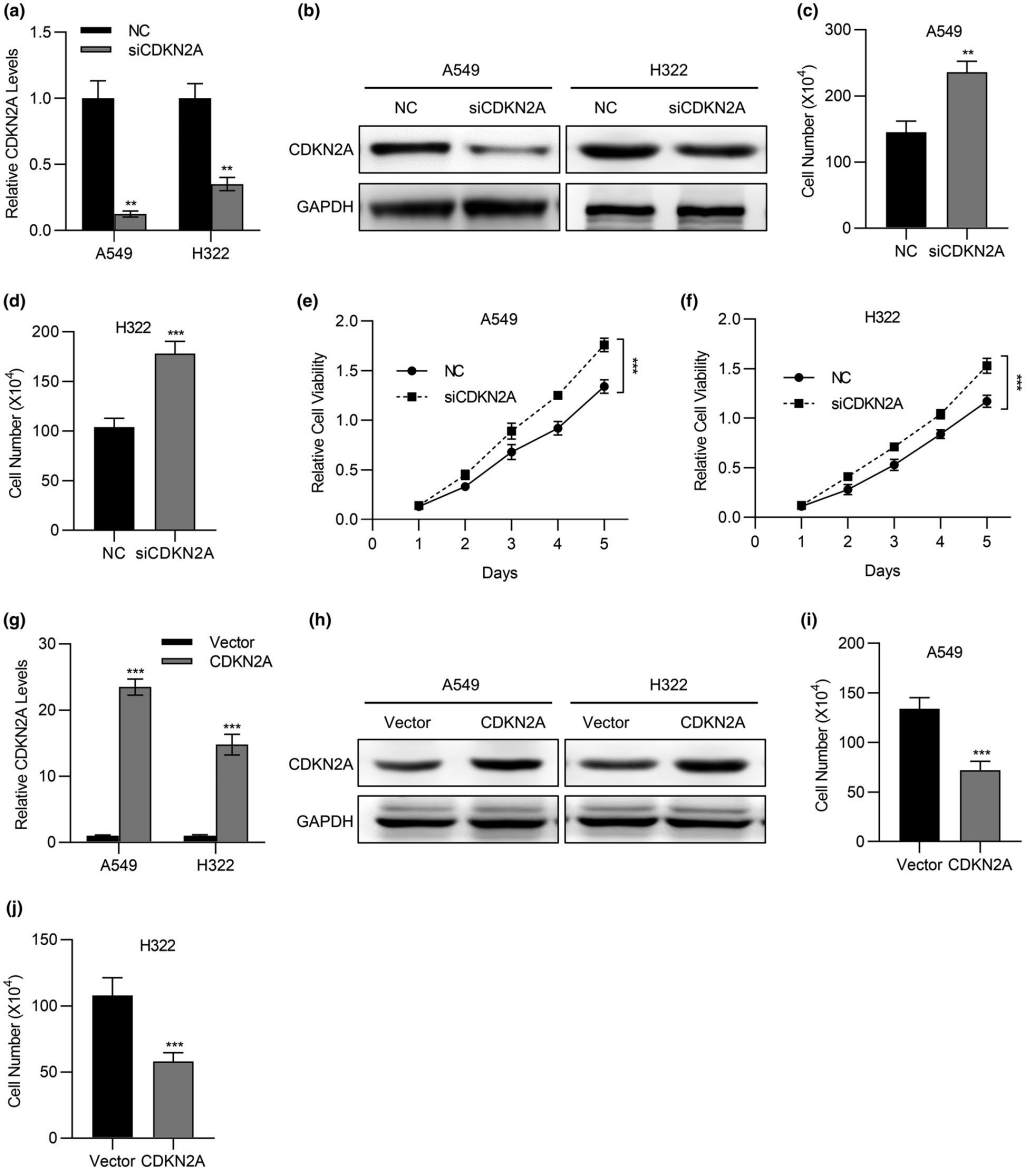
Loss of CDKN2A promoted lung cancer proliferation. (a) The mRNA expression of CDKN2A in A549 and H322 cells transfected with CDKN2A siRNA was determined by qPCR. (b) The expression of CDKN2A in A549 and H322 cells transfected with CDKN2A siRNA was determined by western blot. (c and d) Cell viability of A549 cells (c) or H322 cells (d) transfected with CDKN2A siRNA was determined by cell count assay. (e and f) Cell viability of A549 cells (e) or H322 cells (f) transfected with CDKN2A siRNA was determined by MTT assay. (g) The mRNA expression of CDKN2A in A549 and H322 cells transfected with CDKN2A expressing plasmid was determined by qPCR. (h) The expression of CDKN2A in A549 and H322 cells transfected with CDKN2A expressing plasmid was determined by western blot. (i and j) Cell viability of A549 cells (h) or H322 cells (i) transfected with CDKN2A expressing plasmid was determined by cell count assay. Data are shown as mean ± SD. *p < 0.05; **p < 0.01; ***p < 0.001; ns, not significant

Our previous analysis suggested the potential tumor suppressor role of *CDKN2A* in lung cancer, which prompted us to clarify this in cell culture. To this end, we specifically silenced *CDKN2A* with siRNA in A549 and H322 cells. The success in knockdown of *CDKN2A* gene was confirmed at both transcript (Figure 3a) and protein levels (Figure 3b). Cell proliferation was significantly stimulated by *CDKN2A* silencing in both A549 (Figure 3c) and H322 (Figure 3d) cells as indicated by cell counting assay. Consistently, relative cell viability was also increased by *CDKN2A* knockdown in both A549 (Figure 3e) and H322 (Figure 3f) cells as determined by the MTT assay. On the contrary, we established *CDKN2A* overexpression cell lines derived from both A549 and H322 cells (Figure 3g,h), and found that ectopic *CDKN2A* expression greatly inhibited cell proliferation in both cells (Figure 3i,j). Our results suggested that *CDNK2A* exerted antitumor function in lung cancer via inhibiting cell proliferation.

### 3.4 Loss of *CDKN2A* promoted lung cancer migration and invasion

**FIGURE 4:**
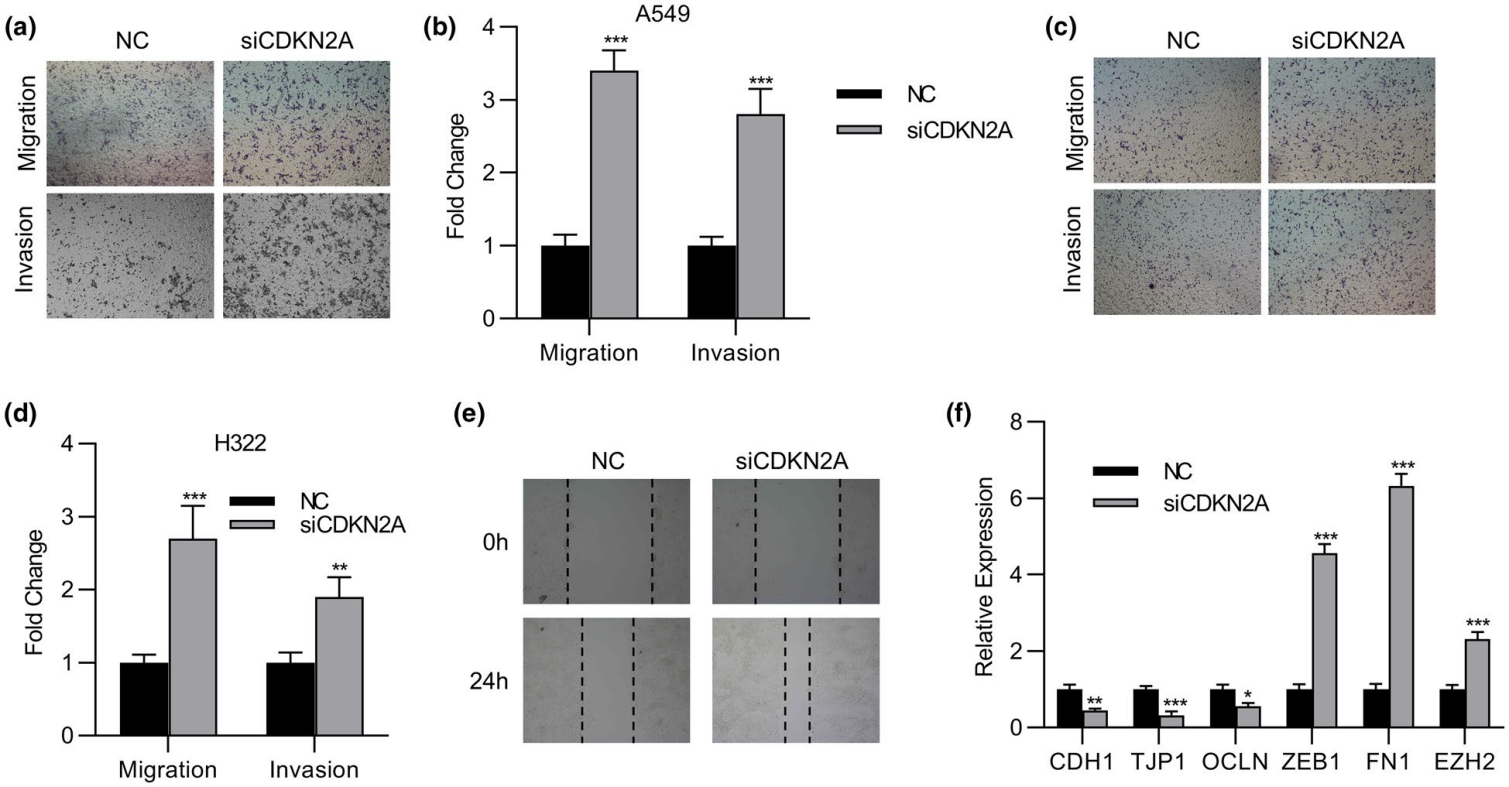
Loss of CDKN2A promoted lung cancer migration and invasion. (a) Transwell of migration and invasion assay of A549 cells transfected with CDKN2A siRNA or NC. (b) The statistic results of the transwell assay in (a). (c) Transwell of migration and invasion assay of the H322 cells transfected with CDKN2A siRNA or NC. (d) The statistic results of the transwell assay in (c). (e) Wound healing assay of A549 cells transfected with CDKN2A siRNA or NC. (f) qPCR analysis of the expression of epithelioid markers CDH1, TJP1, and OCLN or the mesenchymal markers ZEB1, FN1, and EZH2 in A549 cells transfected with CDKN2A siRNA or NC. Data are shown as mean ± SD. *p < 0.05; **p < 0.01; ***p < 0.001; ns, not significant

We next sought to clarify the potential effects of *CDKN2A* on cell migrative and invasive behaviors in lung cancer cells. As shown in Figure 4a, siRNA‐mediated knockdown of *CDKN2A* greatly stimulated both cell migration and invasion in A549 cells, with statistical result presented in Figure 4b. The similar observation was noticed in H322 cells as well (Figure 4c,d). The stimulatory action of *CDKN2A* deficiency on cell migration was further interrogated with wound healing assay. As presented in Figure 4e, the gap closure was tremendously accelerated in *CDKN2A*‐deleted A549 cells. In line with the suppressive roles of *CDKN2A* on cell migration and invasion, the molecular profiling showed decreased epithelial markers including *CDH1*, *TJP1*, and *OCLN*, and increased mesenchymal markers including *ZEB1*, *FN1*, and *EZH2* (Figure 4f). Therefore, our data suggested the suppressive roles of *CDKN2A* on both cell migration and invasion in addition to cell proliferation.

### 3.5 *CDKN2A*‐regulated *MTAP* expression

**FIGURE 5:**
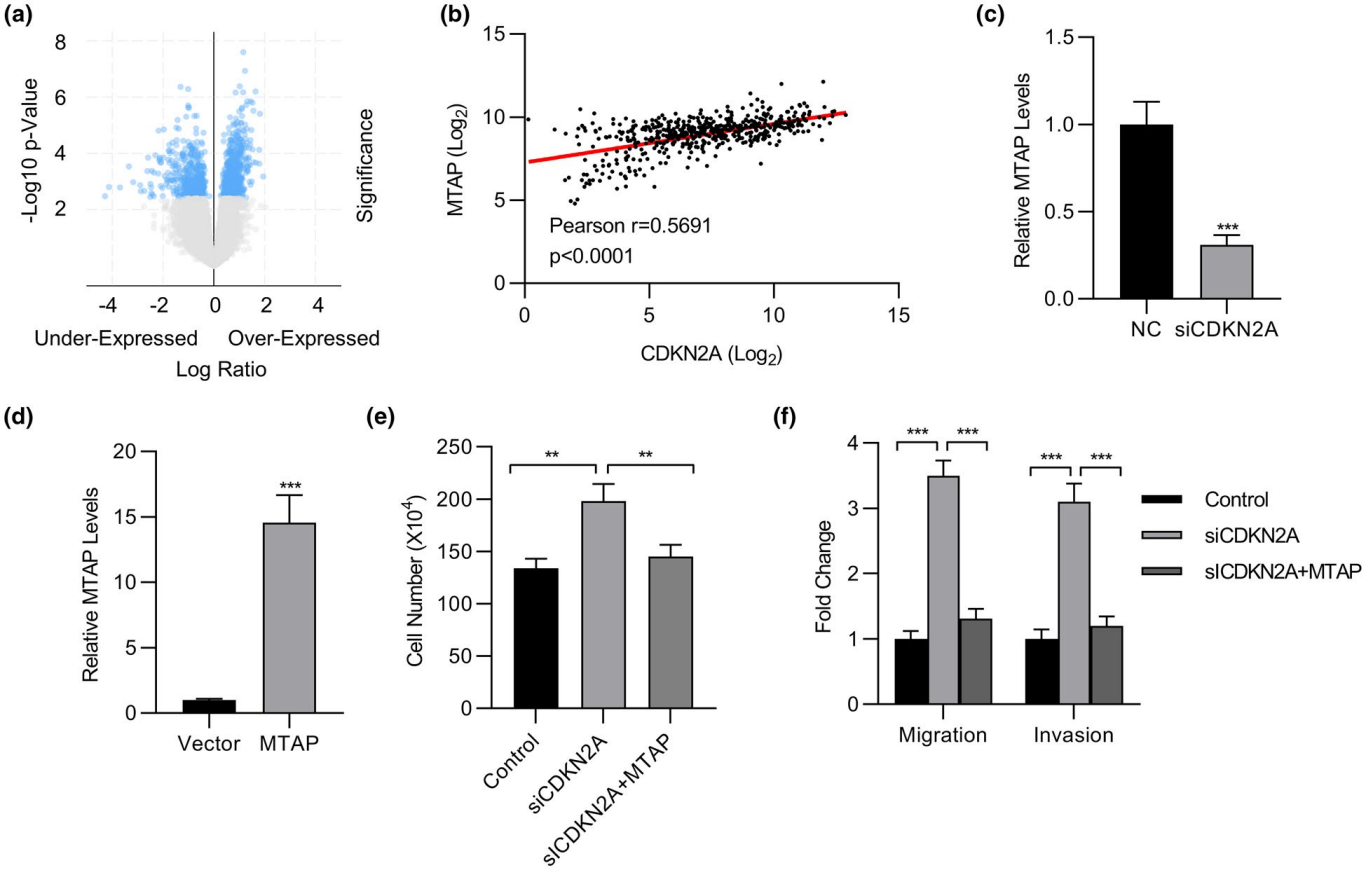
CDKN2A‐regulated MTAP expression. (a) Volcano Plot of the genes correlated with CDKN2A in TCGA dataset. (b) The correlation of MTAP and CDKN2A in TCGA dataset. (c) The MTAP expression levels in A549 cells transfected with CDKN2A siRNA or NC were determined by qPCR. (d) The MTAP expression levels in A549 cells transfected with MTAP expressing plasmid or vector were determined by qPCR. (e) Cell viability of A549 cells transfected with MTAP expressing plasmid and/or CDKN2A siRNA was determined by cell count assay. (f) Transwell of migration and invasion assay of A549 cells transfected with MTAP expressing plasmid and/or CDKN2A siRNA. Data are shown as mean ±SD. *p < 0.05; **p < 0.01; ***p < 0.001; ns, not significant

Next, we sought to understand the molecular mechanism underlying the *CDKN2A*‐stimulated cell proliferative and metastatic phenotype. In view of the nature of CDKN2A as a critical cell cycle‐related protein, alteration of which fundamentally influenced cell cycle progression and expression of an array of genes. Therefore, we analyzed the transcriptome in terms of *CDKN2A* expression status, and the results are presented in Figure 5a as a volcano plot. We identified *MTAP* with high correlation to *CDKN2A* in TCGA dataset (*r* = 0.5691, *p* < 0.0001, Figure 5b). We further demonstrated significant downregulation of *MTAP* transcript in *CDKN2A*‐silenced A549 cells (Figure 5c). To clarify the role of *MTAP* in mediating the antitumoral activities of *CDKN2A*, we then ectopically over‐expressed *MTAP* in *CDKN2A*‐deficient A549 cells (Figure 5d). Cell proliferation stimulated by *CDKN2A* knockdown was completely suppressed by simultaneous overexpression of *MTAP* (Figure 5e). Likewise, both cell migration and invasion that were greatly induced in *CDKN2A*‐deficient A549 were compromised by supplementation with ectopic *MTAP* (Figure 5f). Therefore, our data clearly suggested that *MTAP* predominantly mediated the tumor suppressor roles of *CDKN2A* in lung cancer.

## 4 DISCUSSION

In this study, we systematically analyzed genomic alterations with a specific focus on copy number variation in lung cancer patients. Retrieval of TCGA dataset showed significant deletion in chromosome 9 spanning the *CDKN2A* coding region, which was further identified as one of the most altered genes in multiple datasets. Importantly, lung cancer patients with *CDKN2A* loss manifested relatively poor overall survival and disease‐free survival. The *CDKN2A* transcripts were notably downregulated in patients with genomic depletion. We further demonstrated that ectopic *CDKN2A* expression remarkably inhibited cell proliferation and viability in lung cancer cells, while siRNA‐mediated *CDKN2A* knockdown greatly promoted cell proliferation and viability. In addition, knockdown of *CDKN2A*‐stimulated cell migrative and invasive behaviors, while ectopic introduction of *CDKN2A* significantly compromised the migrative and invasive capacities. Profiling the epithelial‐mesenchymal transition‐related molecular markers demonstrated decreased CDH1, TJP1, and OCLN and increased ZEB1, FN1, and EZH2 in response to *CDKN2A* silencing in lung cancer cells. Mechanistically, we identified *MTAP* as a positively correlated gene with *CDKN2A* in lung cancer, which was significantly downregulated in *CDKN2A*‐deficient cells. Complementation with *MTAP* completely inhibited cell proliferation, migration, and invasion stimulated by *CDKN2A* knockdown. Therefore, our study highlighted the critical contributions of loss of *CDKN2A* in the tumorigenesis and progression of lung cancer, which predominantly depended on downstream *MTAP*. Our study highlighted the antitumor properties of both *CDKN2A* and *MTAP*.

The downregulation of *CDKN2A* was long recognized as a major player in the tumorigenesis of lung cancer via epigenetic suppression. Bradly et al. (2012) reported that *CDKN2A* promoter hypermethylation impacted the outcome in young lung cancer patients. Xiao et al. (2014) suggested the diagnostic values of *CDKN2A* methylation in exhaled breath condensate for early detection of NSCLC. Tuo et al. (2018) proposed *CDKN2A* promoter methylation as a valuable biomarker for NSCLC as well via meta‐analysis. In addition, genomic deletion spanning *CDKN2A* in lung cancer patients has been increasingly acknowledged. Chen et al. reported the deletion of both *FHIT* and *CDKN2A* mRNA in biopsy specimens acquired from lung cancer patients via bronchoscopy for diagnostic purposes (Chen et al., 2013). Jiang et al. (2016) showed that coexistence of *CDKN2A* deletions with overactivation of EGFR signified a poorer response to EGFR‐targeting inhibitor in lung adenocarcinoma patients. Panani et al. (2009) demonstrated that numerous abnormalities in chromosome 9 and *CDKN2A* deletion were detected by FISH in NSCLC patients. Andjelkovic et al. (2011) proposed the concurrent alterations of both *CDKN2A* and *PTEN* as potential biomarkers for particular subgroups of NSCLC patients. Our data were in support of the antitumor properties of *CDKN2A*, deletion of which was notably detected in many lung cancer patients, and therefore, suggested a fundamental role in the tumorigenesis of this disease. Along with epigenetic mechanisms, the biallelic inactivation of *CDKN2A* might heavily contribute to lung cancer incidence.

Our results also highlighted the predominant roles of *MTAP* in mediating the antitumoral activities of *CDKN2A* in lung cancer cells. Complementation with *MTAP* completely inhibited cell proliferation, migration, and invasion which was greatly induced in *CDKN2A*‐deficient cells. We provided the direct evidence in support of the regulation of *MTAP* by *CDKN2A*, and *MTAP* was significantly downregulated in *CDKN2A*‐depleted cells. In view of the complex regulatory network involved in cell cycle control elicited by *CDKN2A* deficiency, we hypothesized that *MTAP* might function indirectly and at the downstream of CDKN2A in lung cancer. However, the detailed molecular events underlying the positive correlation between *CDNK2A* and *MTAP* in the context of lung cancer is yet to be defined. The tumor suppressor roles of *MTAP* uncovered here was in line with multiple previous reports. For instance, Basu et al. (2011) showed that transient *MTAP* analog greatly inhibited human lung cancer growth and metastasis in a mouse xenograft model. Su et al. (2014) suggested *MTAP* as an independent prognostic marker for NSCLC and concurrent loss of both *MTAP* and *CDKN2A* expression indicated more unfavorable prognosis. Schmid et al. (1998) characterized that homozygous deletion of *MTAP* in primary NSCLC was more frequent than *CDKN2A*. This observation was further validated in multiple forms of human cancers, which was suggested to confer heavy dependence on the PRMT5 arginine methyltransferase activity in cancer cells (Kryukov et al., 2016). Interestingly, Mavrakis et al. (2016) discovered that viability of *MTAP*‐deficient cancer cells was impaired by depletion of PRMT5. Since *MTAP* is frequently deleted in human cancers due to its chromosomal proximity to *CDKN2A*, it was also speculated that inhibitors of PRMT5 could be utilized in potential therapy for *MTAP*/*CDKN2A*‐deleted tumors (Mavrakis et al., 2016). In gastrointestinal stromal tumors, Huang et al. suggested that homozygous deletion of *MTAP* as a poorer predictor in clinical outcome (Huang et al., 2009). Cheng et al. (2017) demonstrated that deletion and downregulation of *MTAP* led to the motility of esophageal squamous carcinoma cells.

## 5 CONCLUSION

In summary, here, we have discovered that CDKN2A loss promoted lung cancer progression and correlated with poor survival outcomes in lung cancer, and consolidated the role of CDKN2A‐MTAP signaling in the context of lung cancer, which might offer novel therapeutic and prognostic opportunities clinically against this disease.

## CONFLICT OF INTERESTS

The authors declare that they have no competing interests.

## AUTHOR CONTRIBUTIONS

W.L, C.Z, T.H, S.Y, M.Z^1^, Y.J conducted the experiments and analyzed the data; W.L, B.L wrote the manuscript; B.L conceived and supervised the study.
